# Long-Term Running Exercise Delays Age-Related Changes in White Matter in Rats

**DOI:** 10.3389/fnagi.2020.590530

**Published:** 2020-10-23

**Authors:** Lin Chen, Feng-lei Chao, Wei Lu, Lei Zhang, Chun-xia Huang, Shu Yang, Xuan Qiu, Hao Yang, Yuan-yu Zhao, San-rong Wang, Chen Li, Yong Tang

**Affiliations:** ^1^Department of Histology and Embryology, Chongqing Medical University, Chongqing, China; ^2^Affiliated Brain Hospital of Guangzhou Medical University, Guangzhou Huiai Hospital, Guangzhou, China; ^3^Laboratory of Stem Cell and Tissue Engineering, Chongqing Medical University, Chongqing, China; ^4^Department of Pediatrics, Navy General Hospital, Beijing, China; ^5^Department of Histology and Embryology, Capital Medical University, Beijing, China; ^6^Department of Rehabilitation Medicine and Physical Therapy, Second Affiliated Hospital, Chongqing Medical University, Chongqing, China; ^7^Department of Geriatrics Neurology, The Second Affiliated Hospital of Xi’an Jiaotong University, Xi’an, China

**Keywords:** long-term running exercise, rat white matter, myelinated fibers, oligodendrocytes, capillaries

## Abstract

Running exercise, one of the strategies to protect brain function, has positive effects on neurons and synapses in the cortex and hippocampus. However, white matter, as an important structure of the brain, is often overlooked, and the effects of long-term running exercise on white matter are unknown. Here, 14-month-old male Sprague–Dawley (SD) rats were divided into a middle-aged control group (18-month-old control group), an old control group (28-month-old control group), and a long-term runner group (28-month-old runner group). The rats in the runner group underwent a 14-month running exercise regime. Spatial learning ability was tested using the Morris water maze, and white matter volume, myelinated fiber parameters, total mature oligodendrocyte number, and white matter capillary parameters were investigated using stereological methods. The levels of growth factors related to nerve growth and vascular growth in peripheral blood and the level of neurite outgrowth inhibitor-A (Nogo-A) in white matter were measured using an enzyme-linked immunosorbent assay (ELISA). The present results indicated that long-term running exercise effectively delayed the age-related decline in spatial learning ability and the atrophy of white matter by protecting against age-related changes in myelinated fibers and oligodendrocytes in the white matter. Moreover, long-term running exercise prevented age-related changes in capillaries within white matter, which might be related to the protective effects of long-term exercise on aged white matter.

## Introduction

Using stereological methods, previous studies showed no significant loss of neocortical neurons with human aging (Pakkenberg and Gundersen, 1997; Pakkenberg et al., 2003). In addition, there was no significant loss of neurons in the hippocampus and parahippocampal region with aging in rats (Rapp and Gallagher, 1996; Rapp et al., 2002). However, stereological studies and imaging studies have found that white matter changes with normal aging (Tang et al., 1997; Guttmann et al., 1998; Jernigan et al., 2001). In our previous studies, we found decreased white matter volume and loss of myelinated fibers within white matter in aged human brains and aged rat brains (Tang et al., 1997; Yang et al., 2009). Moreover, we also found a decrease in the number of mature oligodendrocytes with age, leading us to hypothesize that demyelination is the main cause of myelinated fiber loss in aged rat white matter (Li et al., 2009; Chen et al., 2011). Besides in normal aging, Nasrabady et al. (2018) found that the abnormalities of myelin and oligodendrocytes in the white matter were the important changes in Alzheimer’s disease (AD) pathology. The myelin formed by mature oligodendrocytes is widely known to enhance the speed and efficacy of axonal conduction, and demyelination reduces the efficiency of signal transmission in neural circuits and impairs brain function (Peters and Sethares, 2002; Peters, 2009). Recently, it was reported that white matter was involved in executive function and the formation and consolidation of spatial learning and memory ability (Steadman et al., 2020). Thus, white matter changes might be one of the key causes of brain function decline with normal aging.

Unlike neurons in the cortex, the myelin sheaths and oligodendrocytes in the white matter are able to regenerate and may be repaired through drug treatments and behavior interventions. Overwhelming evidence has revealed that physical exercise can prevent the decline in cognition associated with aging (van Gelder et al., 2004; Weuve et al., 2004; Singh-Manoux et al., 2005; Mora and Valencia, 2018; Juarez and Samanez-Larkin, 2019). Furthermore, Marks et al. (2007) and Gons et al. (2013) found that physical activity protected white matter integrity in aged brain. Changes in white matter integrity reflect changes in myelinated fibers in the white matter, and changes in myelinated fibers are related to changes in myelin sheaths and mature oligodendrocytes. In our previous study, using stereological methods, we found that running exercise could prevent demyelination in the white matter of transgenic AD mice (Zhang L. et al., 2017). However, using diffusion tensor imaging (DTI), Clark et al. (2019) found that 6 months of aerobic exercise could not improve cerebral white matter microstructure in older adults. Thus, whether running exercise could delay the changes in myelinated fibers, myelin sheaths, and mature oligodendrocytes in the white matter of the aging brain is unclear.

If running exercise could delay the age-related changes in the white matter of the aging brain, what are the mechanisms underlying exercise-induced changes in white matter? Previous studies have indicated that cerebral capillary density deficits are associated with regional hypoperfusion in aged brains and that a decline in cerebral blood flow is linearly related to age (Riddle et al., 2003; Stoquart-ElSankari et al., 2007). White matter, compared to other brain tissues, has a lower capillary density and is more susceptible to hypoxic–ischemic injury (Anstrom et al., 2002; Thore et al., 2007). In our previous study, we found a marked loss of capillaries in the white matter of rats with aging, which might have important implications for age-related white matter changes and age-related cognitive impairments (Shao et al., 2010). Similarly, Hase et al. (2019) found more vascular pathology changes in aged white matter than young. A large amount of evidence has indicated that exercise has positive effects on brain blood vessels; for example, exercise has been shown to induce angiogenesis and increase cerebral blood volume in the rat primary motor cortex (Swain et al., 2003), increase capillary density and enhance cerebrovascular integrity in the rat striatum (Ding et al., 2006), increase endothelial cell proliferation in the adult rat hippocampus and prefrontal cortex (Ekstrand et al., 2008), and reverse age-related declines in blood vessels in the rat substantia nigra (Villar-Cheda et al., 2009). Murugesan et al. (2012) reported that there was no change in the capillary density of white matter in aged mice after 4 weeks training. In our previous studies, using stereological methods, we found that running exercise remediated the loss of the capillaries in white matter in a rat model of depression and in APP/PS1 transgenic AD mice (Chen et al., 2016; Zhang Y. et al., 2017). In addition, Trigiani et al. (2020) found that exercise could prevent the collapsing capillaries within white matter and reduced CBF induced by a high cholesterol diet. The effect of running on the capillaries in white matter is controversial. Whether long-term running exercise has a protective effect on the capillaries in aged white matter needs to be investigated. In addition, previous studies have shown that exercise could increase brain-derived neurotrophic factor (BDNF) and vascular endothelial growth factor (VEGF) in peripheral blood (Morland et al., 2017; Pedersen, 2019), and exercise-induced high peripheral VEGF and BDNF levels might improve hippocampal perfusion and volumes in older adults (Maass et al., 2016). We speculated that the effect of long-term running exercise on capillaries in white matter might also be related to the growth factors in peripheral blood. However, Nofuji et al. (2008) reported that long-term exercise decreased the BDNF level in peripheral blood. Thus, whether long-term running exercise has a protective effect on the growth factors in peripheral blood of aged rats needs to be investigated.

To address the above issues, 14-month-old Sprague–Dawley (SD) rats were used to study the effects of exercise on aged white matter.

## Materials and Methods

### Animals and Running Procedures

All SD rats were provided by the laboratory animal center of Chongqing Medical University. Rats were housed in a central animal facility with a controlled temperature (22 ± 2°C) and a consistent light/dark cycle (12 h/12 h). Food and water were available *ad libitum*. All procedures conformed to the standards set forth in the National Institutes of Health Guide for the Care and Use of Laboratory Animals (NIH publications No. 85-23).

In total, 30 14-month-old SD male rats were randomly assigned to a middle-aged control group (*n* = 10), an aged control group (*n* = 10), and an aged running group (*n* = 10). The rats in the middle-aged control group were housed in sedentary conditions from 14 to 18 months old (referred to as the 18-month-old control group). The rats in the aged control group were housed in sedentary conditions from 14 to 28 months old (referred to as the 28-month-old control group). The rats in the aged running group underwent treadmill exercise from 14 to 28 months old (referred to as the 28-month-old runner group). A straight six-lane treadmill (600 mm × 95 mm × 115 mm per lane) with electrical stimulation connected to a manual control system (Beijing Sunny Instruments Co. Ltd. China) was used. During the first 2 weeks, the rats were acclimated to running on the treadmill, and the running speed was gradually increased from 5 to 20 m/min for 20 min per day. After the acclimation period, the running speed was maintained at 20 m/min, 20 min per day, 5 days per week for 14 months (Redila and Christie, 2006; Stranahan et al., 2007).

### Spatial Learning Ability Testing

The Morris water maze was used to test the spatial learning ability of each group of rats. The apparatus used for the Morris water maze was a circular pool with a diameter of 1.5 m and an automatic tracking and analysis system (SLY-R01& WMS, Beijing Sunny Instruments Co. Ltd., China). The pool was filled with black water dyed with ink, and the water temperature was controlled at 22 ± 2°C during the experiment.

The rats were trained to swim to a circular platform submerged 1.5 cm beneath the water. The platform was randomly placed into a testing pool, but the platform location was kept constant throughout testing. The overall testing lasted 5 days, with four trials per day. The four starting positions, which were labeled A, B, C, and D at the boundaries of the four different quadrants, were the same on each day. The order of starting positions in the four trials was the same on the same day for all rats. However, the order of starting positions was randomly determined on different testing days. In each trial, the rat was placed into the testing pool at one of four designated starting positions and allowed to swim for a maximum of 180 s until it found the platform and remained on the platform for 15 s. If a rat failed to find the platform within 180 s, it was guided to the platform and stayed on the platform for 15 s. The interval time of adjacent tests for the same rat was no less than 15 min. The time elapsed until the platform was reached (escape latency) was recorded.

### Tissue Processing

After the Morris water maze test was performed, five rats in each group were chosen at random and deeply anesthetized by intraperitoneal injection of 1% pentobarbital sodium (0.4 ml/100 g). Then, the rats were perfusion fixed with 4% paraformaldehyde. After perfusion, the brain was removed and separated from the cerebellum, brain stem, and cranial nerves under the pavimentum cerebri. Right or left hemispheres were randomly selected, postfixed with 2% paraformaldehyde and 2.5% glutaraldehyde for more than 24 h, and used to analyze the white matter volume and the myelinated fibers and capillaries within the white matter. The remaining hemispheres were stored in 4% paraformaldehyde and used to analyze the total number of 2′-3′-cyclic nucleotide 3′-phosphodiesterase-positive cells (CNPase^+^ cells). The other five rats in each group were anesthetized with 1% pentobarbital sodium (0.4 ml/100 g), the heart was exposed, and blood was obtained from the right ventricle. After 1 h, the blood was centrifuged at 2,000 rpm/min for 20 min at room temperature, and the supernatant was carefully collected, stored at −20°C and used for enzyme-linked immunosorbent assay (ELISA). The rats were sacrificed, and the fresh white matter tissue of the brain was isolated. The protein in white matter was extracted by radioimmunoprecipitation assay (RIPA) lysis buffer (P0013K, Beyotime, China) with phenylmethanesulfonyl fluoride (PMSF, ST506, Beyotime, China), stored at −20°C and used for ELISA.

### Estimation of White Matter Volume

**FIGURE 1:**
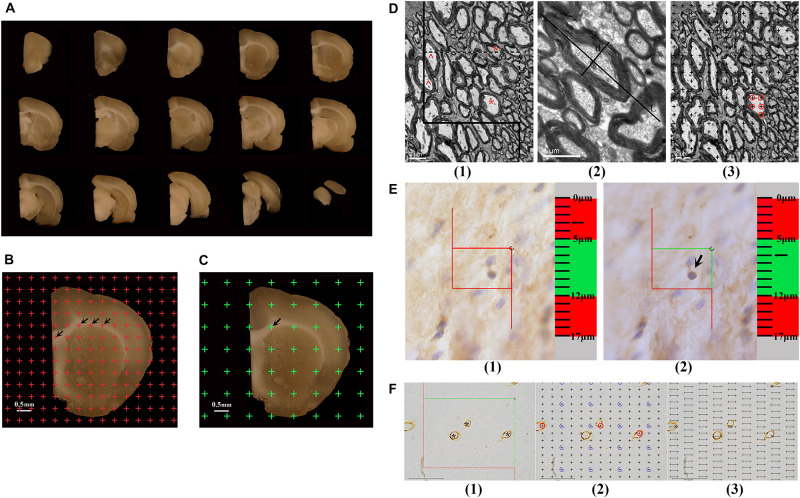
**(A)** The successive 1 mm-thick coronary slabs of one hemisphere. **(B)** For estimating the total volume of the white matter, an equidistant point grid is superimposed at random onto the photograph of the successive 1-mm-thick coronary slabs, and the number of the points hitting the white matter is counted, as indicated by the arrows. Bar = 0.5 mm. **(C)** For sampling the tissue blocks of the white matter, a transparent equidistant point grid is randomly placed on the successive 1-mm-thick coronary slabs, and those points hitting the white matter are sampled. The arrow shows a point hitting the white matter. Bar = 0.5 mm. **(D)** An illustration of the method used to estimate the myelinated fibers in the white matter is shown. **(1)** For estimating the length of the myelinated fibers, an unbiased counting frame is superimposed at random onto the image captured with a transmission electron microscope (TEM). The myelinated fiber profiles are counted if they are completely inside the counting frame or partly inside the counting frame but only touching the counting lines (dotted lines). *Two myelinated fiber profiles that are counted. The myelinated fibers are excluded if they touch the exclusion lines (solid lines). ^Two myelinated fiber profiles that are excluded. Bar = 2 μm. **(2)** The myelinated fiber diameter is measured, as marked by d. L denotes the longest axis of the myelinated fiber. Bar = 1 μm. **(3)** For estimating the volume of the myelinated fibers, a transparent equidistant counting grid is superimposed onto the image captured with TEM. The number of points hitting the myelinated fibers is counted. ◯The points hitting a myelinated fiber. Bar = 2 μm. **(E)** An illustration of the way to count the number of CNPase^+^ cells with the optical disector technique is shown. **(1)** The CNPase^+^ cells in the guard zone are not counted. **(2)** The CNPase^+^ cells with nuclei that are clearly in focus in the counting zone, but not in focus in the guard zone, are counted. The arrow shows the CNPase^+^ cell that is counted. **(F)** An illustration of the way to estimate the capillaries in the white matter is shown. **(1)** For estimating the lengths of capillaries, an unbiased counting frame is superimposed at random onto the image captured with a light microscope. The capillary profiles are counted if they are completely inside the counting frame or partly inside the counting frame but only touching the counting lines (green lines), as indicated by *. The capillary profiles are excluded if they touch the exclusion lines (red lines). Bar = 40 μm. **(2)** For estimating the volume of the capillaries, a transparent equidistant counting grid is superimposed onto the image captured with a light microscope. The number of points hitting the capillary profiles is counted, as indicated by ◯. Bar = 40 μm. **(3)** For estimating the surface area of the capillaries, transparent equidistant test lines are superimposed onto the image captured with a light microscope. The number of intersections between the test lines and capillary luminal surfaces is counted, as indicated by **×**. Bar = 40 μm.

The hemispheres postfixed with 2% paraformaldehyde and 2.5% glutaraldehyde were embedded in 6% agar and cut into 1-mm-thick coronal slabs, starting randomly at the rostral pole. Then, all caudal surfaces of slabs were photographed using a dissecting microscope (Figure 1A). An equidistant point grid was randomly placed on each photograph (Figure 1B). The grid points hitting the white matter were counted. The white matter volume was calculated according to Cavalieri’s principle (Gundersenn et al., 1988; Tang et al., 1997):

(1) V⁢w⁢m= 2×t×a⁢(p)×∑P⁢w⁢m⁢

where *Vwm* indicates the total volume of the white matter, *t* indicates the slab thickness (1 mm), *a(p)* indicates the area associated with each grid point (0.10 mm^2^), and Σ*Pwm* indicates the total number of grid points hitting the white matter per rat.

### White Matter Sampling and Specimen Preparation

The 1-mm-thick coronary slabs used to estimate the parameters of myelinated fibers within the white matter were randomly sampled every two slabs, and the first slab was randomly sampled in the first two slabs. Transparent equidistant points were randomly superimposed on the caudal surface of the sampled slabs (Figure 1C). Based on the points that hit the white matter, six or seven tissue blocks (approximately 1 mm^3^) were randomly sampled per rat. This systematic random sampling provided an equal sampling probability for the white matter (Howard and Reed, 1998). The sampled tissue blocks were fixed with 2% paraformaldehyde and 2.5% glutaraldehyde for 2 h at 4°C; rinsed three times with 0.1 M phosphate-buffered saline (pH 7.2); postfixed with 1% 0.1 M phosphate-buffered osmium tetroxide (OsO_4_) for 2 h at 4°C; gradually dehydrated using 50% ethanol, 70% ethanol, and 90% ethanol solutions, followed by a 90% ethanol and 90% acetone mixture, and a 100% acetone solution; infiltrated with epoxy resin 618 (Chenguang chemical industry, Sichuan, China) for 3 h at 37°C; and embedded in Epon according to the isector technique (Nyenggard and Gundersen, 1992). This technique assures that isotropic, uniform, and random sections are obtained so that the fibers in each direction in three-dimensional space have the same probability of being sampled. Then, the tissue blocks were re-embedded in an oven for 16 h at 37°C, 12 h at 45°C, and 14 h at 60°C. One section with a thickness of 60 nm was cut from each Epon block with an ultramicrotome and viewed with a transmission electron microscope (TEM, Hitachi-7500, Hitachi. Ltd, Japan) at 6,000 × magnification. A set of six fields were randomly sampled and photographed in each section and used to estimate the parameters of myelinated fibers within the white matter.

The remaining 1-mm-thick coronary slabs were used to estimate the parameters of capillaries in the white matter. Eight or nine tissue blocks (approximately 3–4 mm^3^) were randomly sampled per rat as above and dehydrated three times in 30% sucrose solutions. Then, according to the principle of the isector technique, the tissue blocks were embedded with optimal cutting temperature (OCT) compound (SAKURA, 4583, Japan) and sectioned at 6 μm in the parallel direction using a freezing microtome (LEICA CM1950, Germany), and the process was repeated four times in different directions. Collagen IV was used to mark the basement membrane of the vessel. These sections were microwave-heated in citric acid buffer solution for 15 min; soaked in 3% H_2_O_2_ for 10 min at room temperature; successively incubated with normal goat serum for 30 min at 37°C, anti-rat collagen IV antibody (ab6586; Abcam, Cambridge, United Kingdom) diluted to 1:300 for 8 h at 4°C, secondary antibody solution (biotinylated goat-anti-rabbit IgG) for 30 min at 37°C, and S-A/HRP for 30 min at 37°C; and finally developed in diaminobenzidine (DAB, ZLI-9032, ZSGB; Beijing, China) for approximately 10 min. Then, the sections were observed with light microscopy at 1,000 × magnification. A set of three to six fields in each section were randomly sampled and photographed and used to estimate the parameters of capillaries within the white matter. Vessels with diameters less than 10 μm were defined as capillaries (Alba et al., 2004).

### Sampling of Serial Tissue Sections and Immunohistochemical Staining

The remaining hemispheres, stored in 4% paraformaldehyde and used to analyze the total number of CNPase^+^ cells, were successively dehydrated for 24 h in 10% sucrose, 20% sucrose, and 30% sucrose. After dehydration, the hemispheres were embedded in OCT compound (SAKURA, 4583, United States) and then coronally sliced at 50-μm equidistant intervals with a cryo-ultramicrotome (Leica Microsystems, CM1950, Germany), starting randomly at the rostral pole. One section was sampled every 20 sections, and the first section was sampled randomly from the first 20 sections. Therefore, the section sampling fraction (ssf) was 1/20. On average, 14–15 sections were sampled per rat brain. The sampled serial tissue sections were labeled with the anti-CNPase antibody. These sections were treated with acetone for 30 min at room temperature; soaked in 3% H_2_O_2_ for 1 h at room temperature; successively incubated with 10% normal goat serum for 1 h at 37°C, anti-mouse CNPase antibody (SAB4200693; Sigma, United States) diluted to 1:900 for 72 h at 4°C, secondary antibody solution (biotinylated goat-anti-mouse IgG) for 2 h at 37°C, and S-A/HRP for 2 h at 37°C, developed in diaminobenzidine (DAB, ZLI-9032, ZSGB; Beijing, China) for approximately 10 min; and counterstained with Mayer’s hematoxylin to label nuclei. Then, the sections were analyzed with the Visiopharm Image Analysis & Stereology System (VIS, New CAST, Denmark), and the total number of CNPase^+^ cells in the white matter was counted with the optical disector technique.

### Enzyme-Linked Immunosorbent Assay

The protein concentrations were measured by a bicinchoninic acid (BCA) protein assay kit. Then, the levels of growth factors related to nerve growth and vascular growth in peripheral blood, such as BDNF, nerve growth factor (NGF), vascular endothelial growth factor A (VEGF), insulin-like growth factor 1 (IGF-1), and fibroblast growth factor 1 (FGF1), were measured with a Rat BDNF PicoKine ELISA kit (EK0308, BOSTER, China), Rat NGF/NGF beta PicoKine ELISA kit (EK0471, BOSTER, China), Rat VEGF PicoKine ELISA kit (EK0540, BOSTER, China), Rat IGF-1 PicoKine ELISA kit (EK0377, BOSTER, China), and Rat FGF1 PicoKine ELISA kit (EK1173, BOSTER, China), respectively. The level of Neurite outgrowth inhibitor-A (Nogo-A) was measured with a Rat Nogo-A ELISA (RAB1919, Sigma-Aldrich, United States).

### Estimation of Myelinated Fibers in the White Matter

#### The Length Density and Total Length of Myelinated Fibers in the White Matter

An unbiased counting frame was randomly superimposed onto each photograph captured by TEM (Figures 1D1,2). The number of myelinated fiber profiles was counted according to forbidden line rules (Figure 1D1) (Gundersenn et al., 1988), and the diameters of myelinated fibers were measured as previously described (Figure 1D2) (Tang et al., 1997). The length density of myelinated fibers in the white matter, *Lv(mf-wm)*, was calculated according to the following formula (Tang et al., 1997):

(2) $$L\,v\,\left(m\,f-w\,m\right)=2\times \,\frac{\sum Q\,\left(m\,f\right)}{\sum A\,\left(f\,r\,a\,m\,e\right)}$$

where Σ*Q(mf)* indicates the total number of myelinated fiber profiles counted per rat, and Σ*A(frame)* indicates the total area of counting frames used per rat.

The total length of myelinated fibers in the white matter equaled the length density value of myelinated fibers multiplied by the total white matter volume per rat, *Vwm*.

#### The Volume Density and Total Volume of Myelinated Fibers in the White Matter

A transparent equidistant counting grid was randomly superimposed onto each photograph captured by TEM (Figure 1D3). The number of points that hit the white matter and the number of points that hit the myelinated fibers in the white matter were counted. The volume density of myelinated fibers in the white matter, *Vv(mf-wm)*, was estimated according to the following formula (Tang et al., 1997):

(3) $$V\,v\,\left(m\,f-w\,m\right)=\frac{\sum P\,\left(m\,f\right)}{\sum P\,\left(f\,r\,a\,m\,e\right)}$$

where Σ*P(mf)* indicates the total number of points hitting the myelinated fibers in the white matter per rat, and Σ*P(frame)* indicates the total number of points hitting the white matter per rat.

The total volume of myelinated fibers in the white matter equaled the volume density value of myelinated fibers multiplied by the total white matter volume per rat, *Vwm*.

#### Estimation of the Number of CNPase^+^ Cells in the White Matter

2′3′-Cyclic nucleotide 3′-phosphodiesterase (CNPase) is the myelin-specific protein synthesized by oligodendrocytes, which gradually increases in expression during oligodendrocyte maturation (Jahn et al., 2009). CNPase accounts for about 4% of the total CNS myelin proteins, and it can therefore reflect the maturation and myelination of oligodendrocytes. In this study, the number of CNPase^+^ cells in the white matter was counted with the optical disector technique. The contour of the white matter was traced and delineated using stereological analysis software (New CAST, Denmark) at 4 × magnification. The size of the counting frame was set to 300 μm^2^, the area sampling fraction (asf) was set to 3%, the height of the guard zone was set to 5 μm, and the height of the counting zone was set to 7 μm. The numbers of CNPase^+^ cells in the white matter from each section were counted at 100 × magnification (N.A. 1.40) (Hao et al., 2003; Zhao et al., 2013) (Figure 1E). The average thickness of each section during counting was measured at approximately 17 μm. Therefore, the thickness sampling fraction (tsf) was 7/17 = 0.412. The total number of CNPase^+^ cells in the white matter per rat was estimated using an optical fractionator (Gundersenn et al., 1988; Gundersen et al., 1999; Hao et al., 2003; Zhao et al., 2013) as follows:

(4) $$N=\sum Q\times \frac{1}{s\,s\,f}\times \frac{1}{a\,s\,f}\times \frac{1}{t\,s\,f}$$

where Σ*Q* is the total number of CNPase^+^ cells in the white matter per rat, *ssf* is the section sampling fraction, *asf* is the area sampling fraction, and *tsf* is the thickness sampling fraction.

### Estimation of Capillaries in the White Matter

#### The Length Density and Total Length of Capillaries in the White Matter

An unbiased counting frame was randomly superimposed onto each photograph captured with light microscopy (Figure 1F1). The length density of capillaries in the white matter, *Lv(cap-wm)*, was calculated as follows (Gundersenn et al., 1988; Shao et al., 2010; Qiu et al., 2011):

(5) $$L\,v\,\left(c\,a\,p-w\,m\right)=2\times \frac{\sum Q\,\left(c\,a\,p\right)\,}{\sum A\,\left(f\,r\,a\,m\,e\right)\,}$$

where Σ*Q(cap)* indicates the total number of capillaries in the white matter counted per rat, and Σ*A(frame)* indicates the total area of counting frames used per rat.

The total length of capillaries in the white matter equaled the length density value of capillaries multiplied by the total white matter volume per rat, *Vwm* (Gundersenn et al., 1988; Shao et al., 2010; Qiu et al., 2011).

#### The Volume Density and Total Volume of Capillaries in the White Matter

A transparent equidistant counting grid was randomly superimposed onto each photograph captured by light microscopy (Figure 1F2). The number of points that hit the white matter and the number of points that hit the capillaries in the white matter were counted. The volume density of capillaries in the white matter, *Vv(cap-wm)*, was estimated as follows (Gundersenn et al., 1988; Shao et al., 2010; Qiu et al., 2011):

(6) $$V\,v\,\left(c\,a\,p-w\,m\right)=\frac{\sum P\,\left(c\,a\,p\right)\,}{\sum P\,\left(w\,m\right)}$$

where Σ*P(cap)* indicates the total number of points hitting the capillaries in the white matter per rat, and Σ*P(wm)* indicates the total number of points hitting the white matter per rat.

The total volume of capillaries in the white matter equaled the volume density value of capillaries multiplied by the total white matter volume per rat, *Vwm* (Gundersenn et al., 1988; Shao et al., 2010; Qiu et al., 2011).

#### The Surface Area Density and Total Surface Area of Capillaries in the White Matter

Transparent equidistant test lines were randomly superimposed onto each photograph captured with light microscopy (Figure 1F3). The intersection numbers between the test lines and the luminal surface of the capillaries were counted, and the total length of the test lines in the white matter was recorded. The surface area density of capillaries in the white matter, *Sv(cap-wm)*, was estimated according to the following formula (Gundersenn et al., 1988; Shao et al., 2010; Qiu et al., 2011):

(7) $$S\,v\,\left(c\,a\,p-w\,m\right)=2\times \frac{\sum \text{I}\,\left(\text{cap}\right)}{\sum L\,\left(w\,m\right)\,}$$

where Σ*I(cap)* indicates the intersection numbers between the test lines and capillaries, and Σ*L(wm)* indicates the total length of the test lines in the white matter.

The total surface area of capillaries in the white matter equaled the surface area density value of capillaries multiplied by the total white matter volume per rat, *Vwm* (Gundersenn et al., 1988; Shao et al., 2010; Qiu et al., 2011).

### Data Analysis

All data are presented as the mean ± SD. Statistical analyses were performed using SPSS (ver. 19.0, SPSS Inc., Chicago, United States). The Shapiro–Wilk test was used to analyze whether the group means of the data from the Morris water test and the stereological data were normally distributed. Repeated-measures analysis of variance (ANOVA) was used to compare the data from the Morris water test. One-way ANOVA was used to compare the stereological data. The Pearson correlation coefficient was calculated to investigate the relationship between exercise-induced improvement of spatial learning and exercise-induced increase of the white matter, and the myelinated fibers, and capillaries within white matter. A significant difference was considered when *p* < 0.05.

## Results

### Long-Term Treadmill Running Exercise Delays the Decline in Spatial Learning Ability With Aging

**FIGURE 2:**
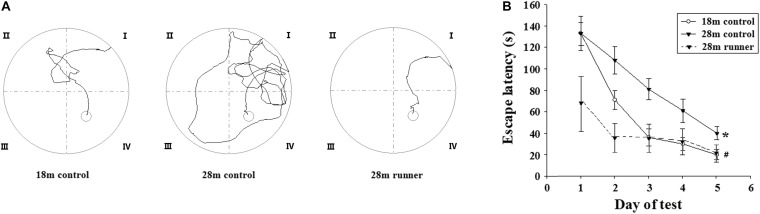
**(A)** Tracked locations of the rats in the Morris water test. The Roman numerals (I,II,III, and IV) represent the first, second, third, and fourth quadrant of the Morris water maze, respectively. **(B)** The mean escape latency in the 18-month-old control group (18 m control), 28-month-old control group (28 m control), and 28-month-old running group (28 m runner). The error bars show the SD. **^∗^***p* < 0.05 when the 28 m control is compared to the 18 m control.**^#^***p* < 0.05 when the 28 m control is compared to the 28 m runner.

Figure 2A shows the traces of the tracked location of the rats in the Morris water maze test. There was no significant difference in the swimming speed among the three groups (0.26 ± 0.04 m/s in the 18 m control group, 0.22 ± 0.03 m/s in the 28 m control group, and 0.25 ± 0.03 m/s in the 28 m runner group). With the increase in training days, the time elapsed for each group to find the platform (escape latency) was gradually decreased (Figure 2B). Compared with the 28-month-old control group, the 18-month-old control group and 28-month-old runner group found the platform more quickly (*p* < 0.05; *p* < 0.05; Figure 2B). However, there was no significant difference in the time to find the platform between the 18-month-old control and 28-month-old runner groups (Figure 2B).

### Long-Term Treadmill Running Exercise Delays the Atrophy of White Matter With Aging

**FIGURE 3:**
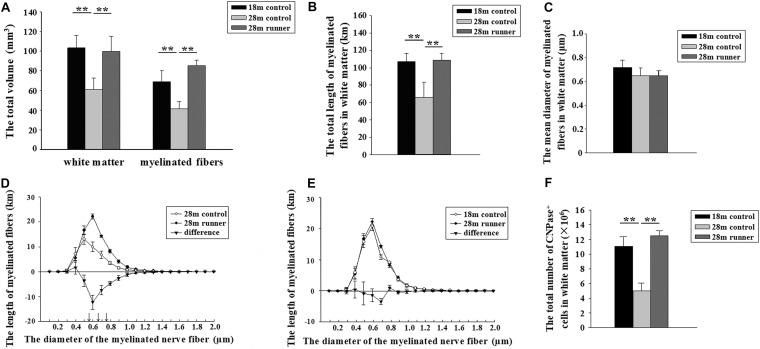
**(A)** The total volume of the white matter and the total volume of myelinated fibers within the white matter of the three groups: the 18-month-old control group (18 m control), 28-month-old control group (28 m control), and 28-month-old runner group (28 m runner). **(B)** The total length of myelinated fibers in the white matter in the three groups. **(C)** The mean diameter of the myelinated fibers in the white matter in the three groups. **(D)** The absolute size distribution on a log scale of the myelinated fiber diameter in the white matter of the 28-month-old control group (◯) and 28-month-old runner group (•). The difference between the two groups is also shown (▼). **(E)** The absolute size distribution on a log-scale of the myelinated fiber diameter in the white matter of the 18-month-old control group (◯) and 28-month-old runner group (•). The difference between the two groups is also shown (▼). **(F)** The total number of CNPase^+^ cells in the white matter of the three groups. The error bars show the SD. **^∗∗^***p* < 0.01. **↑***p* < 0.01.

The 28-month-old control group had a smaller white matter volume compared to the 18-month-old control group (*p* < 0.01; Figure 3A). After running exercise, the total volume of the white matter in the 28-month-old runner group was significantly larger than that in the 28-month-old control group (*p* < 0.01; Figure 3A). However, the white matter volume of the 28-month-old runner group was not different from that of the 18-month-old control group (Figure 3A).

### Long-Term Treadmill Running Exercise Delays the Loss of Myelinated Fibers in White Matter

The 18-month-old control group and 28-month-old runner group had a larger volume in the myelinated fibers of the white matter compared to the 28-month-old control group (*p* < 0.01 and *p* < 0.01; Figure 3A). However, there was no significant difference in the total volume of myelinated fibers within the white matter between the 18-month-old control and 28-month-old runner groups (Figure 3A).

The total length of the myelinated fibers within the white matter was significantly shorter in the 28-month-old control group than that in the 18-month-old control group, but the total length of the myelinated fibers within the white matter of the 28-month-old runner group was significantly longer than that in the 28-month-old control group (*p* < 0.01 and *p* < 0.01; Figure 3B). However, there was no significant difference in the total length of myelinated fibers within the white matter between the 18-month-old control and 28-month-old runner groups (Figure 3B).

There was no significant difference in the mean diameter of myelinated fibers within the white matter among the three groups (Figure 3C).

The total length of the myelinated fibers with a diameter from 0.5 to 0.8 μm was significantly longer in the 28-month-old runner group than in the 28-month-old control group (*p* < 0.01; Figure 3D). However, there were no significant differences between the 18-month-old control group and 28-month-old runner group in the absolute size distribution on a log scale of myelinated fiber diameter (Figure 3E).

### Long-Term Treadmill Running Exercise Delays the Loss of Mature Oligodendrocytes in White Matter With Aging

The 28-month-old control group had significantly fewer CNPase^+^ cells within the white matter than the 18-month-old control group, but the 28-month-old runner group had significantly more CNPase^+^ cells within the white matter than the 28-month-old control group (*p* < 0.01 and *p* < 0.01; Figure 3F). However, there was no significant difference in the total number of CNPase^+^ cells within the white matter between the 18-month-old control group and the 28-month-old runner group (Figure 3F).

### Long-Term Treadmill Running Exercise Delays the Loss of Capillaries in White Matter With Aging

**FIGURE 4:**
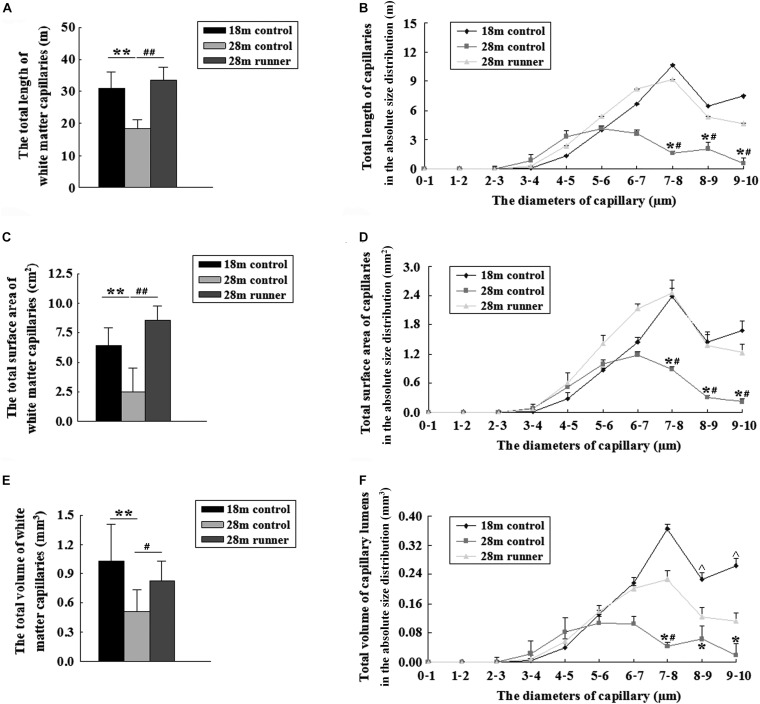
**(A)** The total length of the capillaries in the white matter of the three groups: the 18-month-old control group (18 m control), 28-month-old control group (28 m control), and 28-month-old runner group (28 m runner). **(B)** The absolute distributions of the total length of the capillaries within the white matter of the three groups. **(C)** The total surface area of the capillaries in the white matter of the three groups. **(D)** The absolute distributions of the total surface area of the capillaries within the white matter of the three groups. **(E)** The total volume of the capillaries in the white matter of the three groups. **(F)** The absolute distributions of the total volume of the capillaries within the white matter of the three groups. **^∗^***p* < 0.05 when 28 m control is compared to 18 m control. **^∗∗^***p* < 0.01 when 28 m control is compared to 18 m control.**^#^***p* < 0.05 when 28 m control is compared to 28 m runner. **^##^***p* < 0.01 when 28 m control is compared to 28 m runner. ^*p* < 0.05 when 18 m control is compared to 28 m runner.

The total length of the capillaries within the white matter of the 28-month-old control group was significantly shorter than that of the 18-month-old control group and the 28-month-old control group (*p* < 0.01 and *p* < 0.01; Figure 4A). The total length of the capillaries with a diameter from 7 to 10 μm in the 28-month-old control group was significantly shorter than in the 18-month-old control group and the 28-month-old runner group (*p* < 0.05 and *p* < 0.05; Figure 4B). However, the total length of the capillaries and the absolute size distribution on a log scale of capillary diameter within the white matter of the 18-month-old control group was similar to that of the 28-month-old runner group (Figures 4A,B), which indicated that the decrease in capillary length of white matter in the 28-month-old control group was mainly due to the loss of medium- and large-diameter capillaries (7–10 μm) and that running exercise could increase the medium- and large-diameter capillaries.

Compared with the 28-month-old control group, the 18-month-old control group and 28-month-old runner group had a larger surface area of the capillaries within the white matter (*p* < 0.01 and *p* < 0.01; Figure 4C). The total surface areas of the capillaries with a diameter from 7 to 10 μm in the 28-month-old control group were significantly smaller than those in the 18-month-old control group, and those in the 28-month-old runner group were significantly larger than those in the 28-month-old control group (*p* < 0.05 and *p* < 0.05; Figure 4D). However, there was no significant difference between the 18-month-old control and 28-month-old runner groups in the total surface area of the capillaries within the white matter and in the absolute size distribution on a log scale of capillary diameter (Figures 4C,D). The results indicated that the decrease in capillary surface area of white matter in the 28-month-old control group was mainly due to the loss of medium- and large-diameter capillaries (7–10 μm) and that running exercise could increase the medium- and large-diameter capillaries.

The total volume of the capillaries within the white matter of the 28-month-old control group was significantly smaller than that of the 18-month-old control group and the 28-month-old runner group (*p* < 0.01 and *p* < 0.05; Figure 4E). However, there were no significant differences in the total volume of capillaries within the white matter between the 18-month-old control group and the 28-month-old runner group (Figure 4E). The total volume of capillaries with a diameter from 7 to 10 μm was significantly smaller in the 28-month-old control group than in the 18-month-old control group (*p* < 0.05; Figure 4F). The total volume of capillaries with diameters from 8 to 10 μm was significantly smaller in the 28-month-old runner group than in the 18-month-old control group (*p* < 0.05), and the total volume of capillaries with diameters from 7 to 8 μm was significantly larger in the 28-month-old runner group than in the 28-month-old control group (*p* < 0.05; Figure 4F). All the results indicated that the decrease in capillary volume of white matter in the 28-month-old control group was mainly due to the loss of medium- and large-diameter capillaries (7–10 μm). However, running exercise could only increase the medium diameter capillaries (7–8 μm) within the white matter.

### Correlations Between Behavioral Tests and Stereological Measurements

As shown in Table 1, the exercise-induced behavioral improvement was significantly correlated with the exercise-induced increases in the white matter volume, the length and volume of myelinated fibers within the white matter, and the length, surface area, and volume of the capillaries within the white matter. Moreover, the exercise-induced increases in the white matter volume and the length and volume of myelinated fibers within the white matter were significantly correlated with the exercise-induced increases in the length, surface area, and volume of the capillaries within the white matter.

**TABLE 1:** Correlations between behavioral tests and stereological measurements.

	M (escape latency)	V (wm)	L (mf)	V (mf)
M (escape latency)		*r* = −0.661* *p* = 0.007	*r* = −0.735* *p* = 0.002	*r* = −0.751* *p* = 0.001
L (cap)	*r* = −0.578* *p* = 0.024	*r* = 0.901* *p* < 0.001	*r* = 0.924* *p* < 0.001	*r* = 0.899* *p* < 0.001
S (cap)	*r* = −0.659* *p* = 0.008	*r* = 0.776* *p* = 0.001	*r* = 0.846* *p* < 0.001	*r* = 0.818* *p* < 0.001
V (cap)	*r* = −0.653* *p* = 0.008	*r* = 0.669* *p* = 0.006	*r* = 0.633* *p* = 0.011	*r* = 0.547* *p* = 0.044

M (escape latency) is the mean value of the escape latencies in Morris water maze; V (wm) is the volume of white matter; L (mf) is the length of myelinated fibers within white matter; V (mf) is the volume of myelinated fibers within white matter; L (cap) is the length of capillaries within white matter; S (cap) is the length of capillaries within white matter; V (cap) is the volume of capillaries within white matter.*Significance.

### Long-Term Treadmill Running Exercise Increases Brain-Derived Neurotrophic Factor in the Peripheral Blood of Aged Rats and Decreases the Level of Nogo-A in Aged White Matter

There were no significant differences in the levels of FGF, VEGF, IGF-1, or NGF in the peripheral blood between the 28-month-old control group and the 28-month-old runner group. However, the level of BDNF in the peripheral blood was significantly higher in the 28-month-old runner group than in the 28-month-old control group (*p* < 0.01; Table 2).

**TABLE 2:** The levels of growth factors related to nerve growth and vascular growth.

	Peripheral blood					White matter
		
	Fibroblast growth factor 1 (FGF1) (ng/L)	Vascular endothelial growth factor (VEGF) (pg/L)	Brain-derived neurotrophic factor (BDNF) (ng/L)	Insulin-like growth factor (IGF-1) (ng/L)	Nerve growth factor (NGF) (ng/L)	Neurite outgrowth inhibitor-A (NOGO-A) (pg/mg)
28 m control	684.4 ± 46.3	99.4 ± 10.8	124.9 ± 10.3	34.8 ± 2.0	490 ± 38.8	925.4 ± 131.2
28 m runner	670.3 ± 41.3	103.1 ± 8.5	154.0 ± 8.7^∗∗^	35.7 ± 2.1	472.4 ± 32.7	334.9 ± 61.3^∗∗^

*^∗∗^*p* < 0.01.*

Finally, white matter NOGO-A levels were significantly lower in the 28-month-old runner group than in the 28-month-old control group (*p* < 0.01; Table 2).

## Discussion

Brain function diminishes with increasing age (Watson et al., 2010; Thorvaldsson et al., 2011; Singh-Manoux et al., 2011). As early as Gallagher and Pelleymounter (1988) reported spatial learning ability deficits in aged rats. In the current study, 28-month-old rats performed worse than the 18-month-old rats in the Morris water maze test (escape latency), indicating that the spatial learning ability of rats declined with aging. Overwhelming evidence has revealed that physical exercise can prevent the decline in brain functions associated with aging (Mora and Valencia, 2018; Juarez and Samanez-Larkin, 2019). Running exercise is widely accepted as a simple and affordable method of physical activity. In the current study, 14-month-old male SD rats were subjected to a running protocol for 14 months. We found that the escape latency of the 28-month-old runner group was significantly shorter than that of the 28-month-old control group. Moreover, there were no significant differences in the escape latency between the 18-month-old control group and the 28-month-old runner group. Our results strongly indicate that long-term treadmill exercise could delay the age-related decline in spatial learning ability.

A growing body of research suggests that spatial learning ability is associated with myelin (Wu et al., 2018; Hasan et al., 2019). Myelin is widely known to enhance the speed and efficacy of axonal conduction, and demyelination reduces the efficiency of signal transmission in neural circuits and impairs brain function (Peters and Sethares, 2002; Peters, 2009). In our previous studies, we also found that the decline in spatial learning ability with aging in rats was associated with age-related changes in myelinated fibers (Lu et al., 2014). Myelinated fibers and myelin are distributed in multiple brain regions. The standard view is that spatial learning ability is closely related to the hippocampus, but white matter, as the largest area of myelin distribution, is often overlooked. The latest research reported that water maze learning was related to oligodendrogenesis and *de novo* myelination in the cortex and associated white matter tracts, and preventing oligodendrogenesis could impair memory consolidation of the water maze (Steadman et al., 2020), which indicated that oligodendrocytes and myelin sheaths in white matter were involved in the formation and maintenance of spatial learning and memory. Using stereological methods, Pakkenberg and Gundersen (1997) found that there was no significant neocortical neuron loss with aging, but stereological studies and imaging studies have indicated that white matter changes with normal aging (Tang et al., 1997; Guttmann et al., 1998; Jernigan et al., 2001; Stadlbauer et al., 2012). In our previous studies, we found decreased white matter volume and loss of myelinated fibers within the white matter of aged human brains and aged rat brains (Tang et al., 1997; Yang et al., 2009). Oligodendrocytes, a very important component of white matter, make up approximately 75% of neuroglial cells in subcortical white matter (Moris and Leblond, 1970; Sandell and Peters, 2003). The myelinated fibers in the central nervous system are formed by axons, which are wrapped by oligodendrocytes. Therefore, the structural and functional integrity of oligodendrocytes is important for the myelin sheath and myelinated fibers in the brain white matter. Moreover, oligodendrocytes are vulnerable to injury, particularly in demyelinating diseases (Smith and Lassmann, 2002). In our previous studies, we found fewer mature oligodendrocytes and more demyelination in the white matter of aged rat brains (Li et al., 2009; Chen et al., 2011). In the current study, we found that the total volumes of white matter and myelinated fibers within the white matter, the total length of myelinated fibers, and the total number of oligodendrocytes within the white matter of rats were reduced with age, strongly indicating that there were significant age-related white matter changes.

We found that long-term running exercise delayed the spatial learning ability decline in aged rats. Therefore, we sought to examine whether running exercise could delay the age-related changes in white matter. In a previous study using an imaging analysis method, Colcombe et al. (2006) found that older adults who participated in aerobic fitness training had a larger white matter volume than those who did not participate in aerobic fitness training. In the current study, using the stereological method, we found that after 14 months of long-term running exercise, the total white matter volume in the exercise group was significantly larger than that in the same age group that was not exposed to the exercise protocol, clearly indicating that long-term running exercise could delay the atrophy of the white matter in the aged rat brain. Using DTI, Marks et al. (2007) found that exercise might protect the white matter integrity against aging in select brain regions, and Scholz et al. (2009) found evidence for training-related changes in the white matter structure. However, using the same means, Clark et al. (2019) found that 6 months of aerobic exercise could not improve cerebral white matter microstructure in older adults. The DTI technique is mainly based on random motion of water molecules in the axons of myelinated fibers and can only indirectly, but not accurately, reflect changes in the myelinated fibers in the white matter. In the current study, using unbiased stereological methods, we quantified the parameters of myelinated fibers in the white matter and found that after 14 months of long-term running exercise, the total volume and total length of myelinated fibers within white matter in the exercise group were significantly larger than those in the same age group not exposed to the exercise protocol. Our results indicate that long-term running exercise could prevent the loss of myelinated fibers in the white matter of aged rat brains. Our previous studies showed that small-diameter myelinated fibers might contribute to the significant age-related loss of myelinated fibers in both the human brain and rat brain (Tang et al., 1997; Yang et al., 2009). Therefore, we further investigated the mean diameter of myelinated fibers within the white matter of the rat brain and the absolute size distribution on a log scale of myelinated fiber diameter. We found that running exercise had no significant effects on the mean diameter of myelinated fibers within the white matter of aged rat brains. Moreover, after 14 months of long-term running exercise, the total length of myelinated fibers with a diameter from 0.5 to 0.8 μm in the exercise rats was significantly longer than that in the rats of the same age not exposed to exercise. Our results suggest that long-term running exercise could prevent the loss of small-diameter myelinated fibers in the white matter of aged rat brains. Taken together with the above results, our observations demonstrated that long-term running exercise could delay the atrophy of white matter and prevent the age-related loss of myelinated fibers in the white matter of aged rat brains. In our previous study, we found that running exercise could prevent demyelination in the white matter of transgenic AD mice (Zhang L. et al., 2017). Our current results, together with our previous findings, indicated that running exercise could protect the white matter microstructure both in normal aging and in age-related degeneration diseases.

In the central nervous system, myelin is formed by oligodendrocytes. Exercise has been reported to positively influence oligodendrogenesis in the intact spinal cord and increase the number of oligodendrocytes in the spinal cord injury area (Oh et al., 2009; Krityakiarana et al., 2010). In the present study, we found that after 14 months of long-term running exercise, the total number of mature oligodendrocytes within the white matter in the exercise group was significantly larger than that in the group of the same age not exposed to exercise. We speculated that long-term running exercise might protect against the age-related loss of myelinated fibers in the white matter by preventing the degeneration of oligodendrocytes in the white matter. Recently, it has been reported that running exercise also protects oligodendrocytes in other model animals. Graham et al. (2019) found that exercise increased the oligodendrocytes in the white matter of a mouse model of obesity. Recently, our team found that running exercise significantly increased the number of CNPase-positive oligodendrocytes in the medial prefrontal cortex of the chronic unpredictable stress rat model (Luo et al., 2019).

Cerebral blood flow (CBF) decreases with normal aging (Kashimada et al., 1994; Buijs et al., 1998). Small vessel changes are a leading cause of cognitive decline and functional loss in the elderly (Pantoni, 2010). In our previous study, we used collagen IV to mark the basement membrane of the vessel and found that the total length, total volume, and total surface area of the capillaries in the white matter of aged female rat (27 months) brains were significantly lower than those of young female rat (7 months) brains (Shao et al., 2010). In the current study, we also used collagen IV to mark the basement membranes of the vessels and found that the total length, total volume, and total surface area of the capillaries in the white matter of aged male rat (28 months) brains were significantly lower than those of middle-aged male rat (18 months) brains. In addition, we further investigated the absolute distributions of the length, surface area, and volume of capillaries on a log scale of capillary diameter and found that the length, surface area, and volume of capillaries with diameters from 7 to 10 μm in the 28-month-old rat brains were significantly lower than those in the 18-month-old rat brains. These findings further indicated that there were changes in small blood vessels in the white matter of aged rats. Ainslie et al. (2008) demonstrated that exercise could increase CBF and velocity throughout healthy human aging. In addition, a large amount of evidence indicates that exercise has positive effects on angiogenesis, capillary density, and the cerebrovascular integrity of the brain (Ding et al., 2006; Ekstrand et al., 2008; Villar-Cheda et al., 2009). In the current study, after 14 months of long-term running exercise, the total length, total surface area, and total volume of the capillaries within the white matter in the exercise group were significantly larger than those in the non-exercise group of the same age. These results indicate that long-term running exercise could protect the capillaries in the white matter of aged rats. Previous studies showed that the capillary densities in the motor cortex and hippocampus were increased after a period of exercise both in young and aged rodents (Kleim et al., 2002; Swain et al., 2003; Ding et al., 2006; Villar-Cheda et al., 2009; Rhyu et al., 2010; Murugesan et al., 2012). There have been a few studies on the effects of running on capillaries in the white matter of the brain. Murugesan et al. (2012) reported that 4 weeks of running exercise could increase the capillary density within the hippocampus of aged mice, whereas there was no change in the capillary density of the white matter of aged mice after exercise. Our results seem to be inconsistent with those of Murugesan et al. (2012). There might be three reasons for the difference. First, different animals were used. In the study of Murugesan et al. (2012), the C57Bl/6J mice were used, whereas SD rats were used in our study. Second, different intervention times were used. The running regimen was from the ages of 22–24 months and carried out 5 days per week for only 1 month in the study of Murugesan et al. (2012) whereas the running regimen was from the age of 14 months and carried out 5 days per week for 14 consecutive months in our study. Finally, different quantitative methods were used. In the study of Murugesan et al. (2012), they used CD31 to mark the vessels and analyzed the capillary length per unit volume and the number of branch points using a confocal 3-D rendering approach, whereas we used collagen IV to mark the vessels and analyzed the total length, total surface area, and total volume of capillaries using stereological methods. Although short-term running might not have any effect on white matter capillaries in aged mice, long-term running had a protective effect on white matter capillaries in aged rats. Then, we further investigated the absolute distributions of the length, surface area, and volume of the capillaries on a log scale of the capillary diameter. After 14 months of long-term running exercise, the length and surface area of the capillaries with a diameter from 7 to 10 μm and the volume of the capillaries with a diameter from 7 to 8 μm were significantly larger in the exercise group than in the no-exercise group of the same age. A previous study indicated that the density of the smallest arteries increased in the early phase of exercise, with the small arteries diminishing and the larger arterioles increasing as the exercise continued (Bloor, 2005). In our study, the running regimen was carried out for 14 consecutive months, and the volume, length, and surface area of the larger capillaries were significantly increased, which suggested that long-term running exercise may promote angiogenesis in the white matter of aged brains. Taken together, we speculate that the exercise-induced protection of capillaries in the white matter of aged rats might be associated with the protective effects of exercise on the white matter of aged rats.

Nogo-A is a cytokine that inhibits myelination and is expressed in mature oligodendrocytes (Pernet et al., 2008). In previous studies, silencing Nogo-A promoted remyelination in demyelinating diseases (Yang et al., 2010), and deficits in Nogo-A promoted the repair ability of aged brain injury model mice (Marklund et al., 2009). In the current study, the level of Nogo-A in the white matter was significantly lower in the exercise group than in the group of the same age not exposed to exercise. We speculate that running exercise might protect oligodendrocytes and myelinated fibers in the white matter of aged rats by inhibiting the expression of Nogo-A in the white matter. We also investigated the levels of IGF-1, VEGF, NGF, FGF, and BDNF in the peripheral blood of aged rats but found no significant differences in the levels of IGF-1, VEGF, NGF, and FGF in the peripheral blood between the exercise group and the control group of the same age. However, the level of BDNF in the peripheral blood in the exercise group was significantly higher than that in the non-exercise group of the same age. Previous studies have shown that exercise could increase BDNF, VEGF, and IGF-1 in peripheral blood (Maass et al., 2016; Morland et al., 2017; Pedersen, 2019). Thus, with the exception of BDNF, our results appeared to be inconsistent with previous studies. We speculated that long-term running exercise might have a more lasting and effective effect on BDNF in peripheral blood rather than VEGF and IGF-1. Is there a relationship between an increase in BDNF and a decrease in NogoA? Nogo-A can bind to the Nogo-66 receptor (NgR), Josephson et al. (2003) found that kainic acid led to a marked downregulation of NgR mRNA levels in brain during a time when BDNF mRNA was upregulated instead. The relationship between BDNF and NgR seems to be consistent with our study. A previous study found that exercise could increase the CBF in the hippocampus through increasing BDNF in peripheral blood (Maass et al., 2016). In addition, increased CBF in brain could promote the release of BDNF from cerebral endothelial cells (Borror, 2017). Although we did not measure BDNF level in brain in this study, evidences from previous studies suggested that exercise increased the growth factors in the brain (Pedersen, 2019). Previous studies indicated that BDNF could increase the production of nitric oxide acting through its receptor tropomyosin-related kinase B (Biojone et al., 2015). In our previous study, we found that 4 weeks of running exercise increased nitric oxide synthase activity, nitric oxide content, and the capillary parameters in the rat hippocampus, and the protective effect of running exercise on the capillaries was weakened when the synthesis of endogenous nitric oxide was blocked (Qi et al., 2020). Therefore, we speculated that the exercise-induced increase in BDNF in the peripheral blood might promote the production of nitric oxide and angiogenesis, and then increase the release of BDNF from cerebral endothelial cells, thus promoting the regeneration of oligodendrocytes and myelination through inhibiting the NogoA downstream signaling pathway in the white matter of aged rats.

## Conclusion

The present results indicated that long-term running exercise could effectively delay the age-related decline in spatial learning ability and delay the atrophy of white matter through protecting against age-related changes in myelinated fibers and oligodendrocytes in the white matter. Moreover, long-term running exercise was found to prevent age-related changes in the capillaries within the white matter, which might be related to the protective effects of long-term exercise on aged white matter. It should be noted that the lack of a young runner group was the limitation of the study. In our present study, 14 months of moderate and regular running exercise starting at middle-aged rats could have protective effects on the spatial learning ability and white matter of aged rats. The current results suggested that if we keep long-term moderate and regular exercise, even starting at middle-aged time, we could delay the aging progress of our brain.

## Data Availability Statement

All datasets generated for this study are included in the article/supplementary material.

## Ethics Statement

The animal study was reviewed and approved by the Animal Care and Research Committee of Chongqing Medical University.

## Author Contributions

YT and CL contributed to the conception and design of the study. LC and F-lC wrote the manuscript. LC, F-lC, WL, LZ, C-xH, SY, XQ, Y-yZ, and S-rW performed the animal experimental operation and data analysis. All the authors contributed to the article and approved the submitted version.

## Conflict of Interest

The authors declare that the research was conducted in the absence of any commercial or financial relationships that could be construed as a potential conflict of interest.
